# Association of Medicaid expansion with colon cancer care: treatment patterns and survival in non-metastatic cases from state registry-claims data

**DOI:** 10.1007/s10552-025-01983-8

**Published:** 2025-06-23

**Authors:** Kirsten Y. Eom, Weichuan Dong, Richard S. Hoehn, Jeffrey M. Albert, Uriel Kim, Gregory Cooper, Johnie Rose, Jennifer Tsui, Siran M. Koroukian

**Affiliations:** 1 Center for Health Care Research and Policy at The MetroHealth System, Cleveland, OH USA; 2 Population Cancer Analytics Shared Resource, Case Comprehensive Cancer Center, Case Western Reserve University, Cleveland, OH USA; 3 Department of Population and Quantitative Health Sciences, School of Medicine, Case Western Reserve University, Cleveland, OH USA; 4 Center for Community Health Integration, School of Medicine, Case Western Reserve University, Cleveland, OH USA; 5 Division of Surgical Oncology, University Hospitals, Cleveland, OH USA; 6 Department of Internal Medicine, Cedars-Sinai Medical Center, Los Angeles, CA USA; 7 Department of Population and Public Health Sciences, Keck School of Medicine, University of Southern California, Los Angeles, CA USA; 8 Department of Internal Medicine, University Hospitals, Cleveland, OH USA

**Keywords:** Medicaid expansion, Colon cancer, Cancer treatment patterns, Survival, State registry, Medicaid claims data

## Abstract

**Purpose:**

Despite growing research on Medicaid expansion’s impact on cancer outcomes, there remains a critical need for a more nuanced understanding of how expansion affects cancer care and survival. This study assesses whether Medicaid expansion was associated with improved receipt of standard treatment, timely treatment initiation, and overall survival among colon cancer patients, while examining the specific factors influencing these outcomes.

**Methods:**

Using Ohio’s state cancer registry linked with Medicaid records, we analyzed 688 Medicaid-enrolled patients with non-metastatic colon cancer diagnosed between May 2011 and December 2017. We employed multivariable Poisson and Cox proportional hazard regression analyses to evaluate the impact of Medicaid expansion on treatment and survival outcomes, controlling for individual- and area-level factors.

**Results:**

We observed no significant changes in the likelihood of receipt of standard treatment or timely treatment initiation post-expansion vs. pre-expansion, and no significant differences in these outcomes by Medicaid eligibility criteria post-expansion. However, we observed significantly improved survival (hazard ratio, HR 0.49 [0.28, 0.88]) among patients who became newly eligible for Medicaid under the ACA vs. pre-expansion. Patients enrolled emergently (shortly after/upon diagnosis) were more likely to receive standard treatment (risk ratio, RR 1.14 [1.02, 1.27]).

**Conclusions:**

Our findings provide nuanced insights into Medicaid expansion’s impact on colon cancer care, showing that while expansion did not affect treatment measures, it improved survival among newly eligible patients. Higher standard treatment likelihood among emergently enrolled patients suggests complex post-expansion care dynamics. Further research should investigate mechanisms underlying improved survival and develop interventions to enhance treatment quality alongside observed survival benefits.

**Supplementary Information:**

The online version contains supplementary material available at 10.1007/s10552-025-01983-8.

## Introduction

Colorectal cancer (CRC) is the fourth most common cancer and the second leading cause of cancer death in the U.S., despite a steady increase in survival rates for CRC over recent decades [1]. Improved survival among CRC cases can be attributed to increased routine clinical examinations and CRC screening, more accurate staging, and advancements in surgery, chemotherapy, and radiation treatment [2–4]. A crucial factor enabling patients to access these advances has been the expansion of Medicaid coverage in many states under the Affordable Care Act (ACA).

Research into the impact of Medicaid expansion on cancer-related outcomes—such as screening, diagnosis, treatment, and survival—has been growing. Generally, findings suggest increases in guideline-recommended screening, more early-stage diagnoses, higher rates of cancer-directed treatment, and improved survival rates [5–27]. Recent studies have demonstrated the importance of pre-diagnosis Medicaid coverage: pre-diagnosis Medicaid enrollment was associated with lower likelihood of distant-stage disease across multiple cancer types and breast cancer patients enrolled in Missouri Medicaid more than 90 days before diagnosis showed improved survival compared to those enrolled around diagnosis [28, 29]. However, there remains a need for a more nuanced exploration of factors contributing to improved cancer treatment receipt under Medicaid expansion. This level of detail is crucial because the effectiveness of Medicaid expansion in improving cancer care may depend not only on coverage itself, but also on how different pathways to Medicaid coverage and timing of enrollment relative to diagnosis influence treatment patterns and outcomes. Understanding these timing patterns is especially important from a health system perspective, as they directly influence how care delivery must be organized. Healthcare organizations have had to adapt their cancer care delivery systems and processes to effectively serve a rapidly expanding population of newly insured patients with complex care needs.

Our study addresses this gap by utilizing a unique linked dataset of state cancer registry and Medicaid administrative data from Ohio. Unlike commonly used databases such as National Cancer Data Base (NCDB), Health Care Cost and Utilization Project (HCUP), or state registry databases [25, 30, 31], our dataset allows us to precisely determine when patients enrolled in Medicaid relative to their cancer diagnosis, distinguish between traditional Medicaid enrollees and those who gained coverage through ACA expansion, and track changes in individual Medicaid status over time [32]. While focused on a single state, these detailed data provide pragmatic insights into how expanded insurance coverage, along with census tract-level contextual factors such as income, healthcare access, and rurality, affected access to and quality of cancer care. To that end, our study aim is twofold: (1) to assess whether Medicaid expansion was associated with improved treatment and overall survival among these patients and (2) to examine how different pathways to Medicaid coverage under the ACA and timing of Medicaid enrollment affect cancer care outcomes, including receipt of treatment, timely initiation of treatment, and overall survival.

## Methods

### Data sources and study population

We used the 2011–2017 Ohio Cancer Incidence Surveillance System (OCISS) data to identify individuals diagnosed with local or regional colon cancer. These data were linked with Ohio Medicaid administrative data using patient identifiers; details of the linkage process have been described elsewhere [7]. OCISS is a state registry to which all medical providers are required to report all cancer diagnosed or treated in Ohio and has a gold certification from the North American Association of Central Cancer Registries (NAACCR), indicating the registry meets their highest standards for data completeness, accuracy, and timelines [33, 34]. OCISS collects patient demographics (age at diagnosis, biological sex, race/ethnicity, and marital status), tumor characteristics (primary cancer site and stage at diagnosis), data for each treatment modality, including date and type, and vital status at last follow-up [7]. Ohio Medicaid administrative data include Medicaid enrollment files which contain enrollees’ identifiers, demographic information (age, biological sex, and race/ethnicity), Medicaid eligibility category (ACA-expanded or traditional), timing and length of enrollment, and comorbid conditions. For area-level characteristics, we used the 2013–2017 American Community Survey for census tract-level median household income, level of educational attainment, and proportion of uninsured individuals [35]. We used the Area Health Resources File data for county-level health professional shortage areas and rurality of residence [36].

**Fig. 1:**
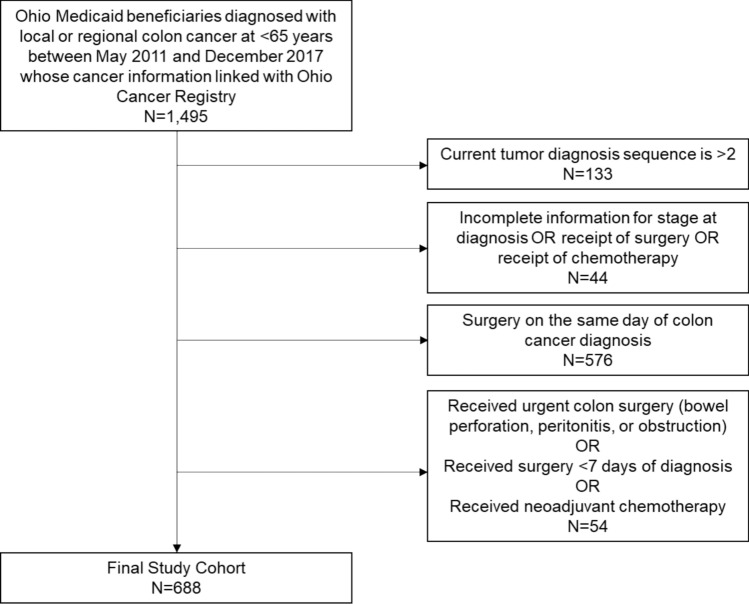
Selection of the study population

Using the linked OCISS and Ohio Medicaid claims data, we identified 1,495 patients diagnosed with local or regional colon cancer between May 2011 and December 2017. Because of high prevalence of colon cancer cases among CRC cases and the complexity and variability of rectal cancer treatment plans [37], we did not include patients with rectal cancer in this study. We also excluded patients diagnosed at a distant stage, as they may not be candidates for curative-intent treatment. The final study population included 688 Medicaid enrollees diagnosed with local or regional colon cancer in Ohio (Fig. 1).

### Outcome measures

The primary outcome measure indicates whether patients received standard treatment within 180 days after cancer diagnosis, determined by NCCN guidelines and expert opinion [38]. For local stage, standard treatment is defined as receipt of surgery. For regional stage, it includes surgery with adjuvant chemotherapy. Patients not receiving standard treatment include those who never received any treatments and those who received partial standard care within 180 days post-diagnosis. We also assessed timely treatment initiation, defined as receiving standard treatment within 30 days following diagnosis [39, 40]. In addition, we used vital status on the last date for which complete death ascertainment was available from OCISS to calculate survival.

### Independent variables

Our primary independent variable is a binary variable distinguishing between pre-expansion (2011–2013) and post-expansion (2014–2017) periods. In the post-expansion period, patients are categorized into the ACA group if they enrolled in Medicaid via new eligibility pathways after the ACA, i.e., childless adults with income < 138% FPL. Patients are categorized into the non-ACA group if they enrolled via eligibility pathways available before the ACA.

### Other individual- and area-level variables

Individual-level variables were selected based on the Andersen Behavioral Model of Healthcare Access and prior literature on cancer care disparities [41–43]. Individual-level variables included age at diagnosis (< 50, 50–54, 55–59, and 60–64), sex (male vs. female), race/ethnicity (non-Hispanic White and other vs. non-Hispanic Black), marital status (not married vs. married), stage at diagnosis (local vs. regional), timing of enrollment with respect to diagnosis (stably enrolled vs. emergently enrolled), and multiple chronic conditions (none, physical conditions only, and other). Due to the small number of Hispanic patients and those from minority groups other than non-Hispanic black, we included them with non-Hispanic white patients. We used the same definition for timing of enrollment with respect to our previous study [7]: we identified patients as stably enrolled if they were continuously enrolled in Medicaid ≥ 4 months prior to diagnosis and patients as emergently enrolled if they were continuously enrolled in Medicaid between 3 months before and after the diagnosis. As for multiple chronic conditions, they were identified from diagnosis codes documented in Medicaid claims data based on Elixhauser comorbidities, grouped as none, physical conditions only, and ‘Other’. The latter category flagged the presence of mental illness or substance use disorders, regardless of their diagnosis of physical conditions.

Area-level variables include census tract-level median household income, percent of adults aged ≥ 25 years with high school diploma, and percent of uninsured adults aged 19–64 years. Each census tract-level variable was operationalized as a categorical variable by quartile. For example, the first quartile includes census tracts with the lowest median household income and the fourth quartile includes census tract with the highest median household income. County-level variables include health professional shortage area and rurality of residence.

### Statistical analysis

We conducted descriptive bivariate analysis using chi-square tests to compare study population characteristics by Medicaid expansion status as well as Medicaid eligibility pathway under the ACA. We employed multivariable Poisson regression with logit link to estimate the impact of Medicaid expansion on cancer care outcomes. This model, which provides robust standard error estimates and interpretable risk ratios, assessed whether patients received standard treatment within 180 days and initiated surgery within 30 days of colon cancer diagnosis, adjusting for individual- and area-level characteristics [44]. We also conducted a multivariable Cox proportional hazard regression analysis to assess the impact of Medicaid on survival among the study population, adjusting for individual- and area-level characteristics except for receipt of treatment. We verified that the Cox proportional hazard assumptions were met. In addition, we repeated these regression analyses to assess the Medicaid expansion by Medicaid eligibility pathway under the ACA (the newly eligible ACA group in the post-expansion vs. pre-expansion; traditional Medicaid group in the post-expansion vs. pre-expansion; ACA vs. pre-expansion).

We identified the timing of enrollment as a potentially significant factor for outcomes and explored Medicaid expansion’s impact on this timing and conducted a sensitivity analysis that excludes timing of enrollment (Supplementary Tables 1 and 2). Given the critical role of stage at diagnosis in prognosis and treatment planning, we also conducted stratified analyses by stage (Supplementary Tables 3 and 4). We compared outcomes between the newly eligible ACA group and the traditional Medicaid (non-ACA) group, acknowledging the inherent differences between these two populations (Supplementary Table 5). All statistical analyses were conducted using R 4.3.2 with a significance level of 0.05, and 95% confidence intervals were calculated for all estimates.

## Results

Our study included 688 patients: 211 in the pre-expansion period and 477 in the post-expansion period, with 300 in the non-ACA group and 177 in the ACA group in the post-expansion group. Table 1 presents the individual- and area-level characteristics of the study population, categorized by Medicaid expansion status and post-expansion Medicaid eligibility criteria group. Receipt of standard treatment and timely initiation of treatment were not statistically different pre- vs. post-expansion and non-ACA vs. ACA groups in post-expansion. Comparing characteristics of Medicaid enrollees pre- vs. post-expansion, the proportion of Non-Hispanic Black patients decreased from 28.4% pre-expansion to 21.0% post-expansion (*p* = 0.041). The timing of enrollment saw a substantial shift (*p* < 0.001), with stably enrolled patients increasing from 61.1% pre-expansion to 75.9% post-expansion, while emergently enrolled patients decreased from 38.9% to 24.1%. We observed additional noteworthy differences comparing non-ACA vs. ACA groups. The ACA group had a higher proportion of 55–59-year-olds and males compared to the non-ACA group (57.1% vs. 47.3%, *p* = 0.050). A higher percentage in the non-ACA than in the ACA group presented with mental health conditions and/or substance use disorders, with or without physical conditions (44.0% compared to 27.7%, *p* < 0.001). The ACA group had a much higher proportion of emergently enrolled patients (40.1% compared to 14.7% in the non-ACA group). Although not statistically significant, a considerably smaller percentage of patients in the ACA than in the non-ACA group resided in areas with the lowest median household income and lowest educational achievement.

**Table 1:** Individual- and area-level characteristics of study population by ACA Medicaid expansion status and by post-expansion Medicaid eligibility criteria

	Pre-expansion	Post-expansion	*p* value	Post-expansion		*p* value
				Non-ACA group^a^	ACA group^b^	
N	211	477		300	177	
*Outcomes measures*						
Receipt of standard treatment^c^	155	348	0.965	210	138	0.074
	(73.5)	(73.0)		(70.0)	(78.0)	
Timely initiation of treatment^d^	128	285	0.887	174	111	0.297
	(60.7)	(59.7)		(58.0)	(62.7)	
*Individual-level characteristics*						
Age at diagnosis (years)			0.292			< 0.001
< 50	57	100		76	24	
	(27.0)	(21.0)		(25.3)	(13.6)	
50–54	44	96		62	34	
	(20.9)	(20.1)		(20.7)	(19.2)	
55–59	53	142		72	70	
	(27.0)	(29.1)		(24.0)	(39.5)	
60–64	57	139		90	49	
	(27.0)	(29.1)		(30.0)	(27.7)	
Male	114	243	0.507	142	101	0.050
	(54.0)	(50.9)		(47.3)	(57.1)	
Non-Hispanic Black	60	100	0.041	71	29	0.077
	(28.4)	(21.0)		(23.7)	(16.4)	
Married	49	134	0.215	81	53	0.558
	(23.2)	(28.1)		(27.0)	(29.9)	
Stage at diagnosis			0.753			0.880
Local	78	184		117	67	
	(37.0)	(38.6)		(39.0)	(37.9)	
Regional	133	293		183	110	
	(63.0)	(61.4)		(61.0)	(62.1)	
Multiple chronic conditions			0.220			0.001
None	54	98		61	37	
	(25.6)	(20.5)		(20.3)	(20.9)	
Physical conditions only	75	198		107	91	
	(35.5)	(41.5)		(35.7)	(51.4)	
Other^e^	82	181		132	49	
	(38.9)	(37.9)		(44.0)	(27.7)	
Timing of enrollment^f^			< 0.001			< 0.001
Stably enrolled	129	362		256	106	
	(61.1)	(75.9)		(85.3)	(59.9)	
Emergently enrolled	82	115		44	71	
	(38.9)	(24.1)		(14.7)	(40.1)	
*Area-level characteristics*						
Median household income			0.296			0.163
Quartile 1 (lowest)	85	162		112	51	
	(40.3)	(34.0)		(37.3)	(28.8)	
Quartile 2	65	149		92	59	
	(30.8)	(31.2)		(30.7)	(33.3)	
Quartile 3	36	107		64	38	
	(17.1)	(22.4)		(21.0)	(21.5)	
Quartile 4 (highest)	25	59		34	29	
	(11.8)	(12.4)		(11.3)	(16.4)	
Percentage of high school diploma among adults aged > 25 years			0.296			0.263
Quartile 1 (lowest)	82	159		108	36	
	(38.9)	(33.3)		(36.0)	(20.3)	
Quartile 2	70	154		95	40	
	(33.2)	(32.3)		(31.7)	(22.6)	
Quartile 3	33	101		86	46	
	(15.6)	(21.2)		(28.7)	(26.0)	
Quartile 4 (highest)	26	63		101	55	
	(12.3)	(13.2)		(33.7)	(31.1)	
Percent of no health insurance among adults aged 19–64 years			0.383			0.626
Quartile 1 (lowest)	35	84		48	36	
	(15.2)	(17.6)		(16.0)	(20.3)	
Quartile 2	58	105		65	40	
	(27.5)	(22.0)		(21.7)	(22.6)	
Quartile 3	51	132		86	46	
	(24.2)	(27.7)		(28.7)	(26.0)	
Quartile 4 (highest)	70	156		101	55	
	(33.2)	(32.7)		(33.7)	(31.1)	
Part or whole county in health professional shortage area	177	417	0.261	266	151	0.355
	(83.9)	(87.4)		(88.7)	(85.3)	
Appalachian/metro status			0.419			0.285
Appalachia	39	109		70	39	
	(18.5)	(22.9)		(23.3)	(22.0)	
Non-Appalachia & non-metro area	20	46		24	22	
	(9.5)	(9.6)		(8.0)	(12.4)	
Non-Appalachia & metro area	152	322		206	116	
	(72.0)	(67.5)		(68.7)	(65.5)	

Frequency (Column %) is presented. Pre-expansion period includes 2011–2013; post-expansion period includes 2014–2017

^a^Non-ACA group includes Medicaid eligibility criteria that were not amended or created as a result of the ACA in 2014

^b^ACA group includes Medicaid eligibility categories that were created and became in effect as a result of the ACA in 2014

^c^Receipt of standard treatment is defined as receipt of standard treatment within 180 days after cancer diagnosis. For local stage, we defined the standard treatment as receipt of surgery. For regional stage, we defined the standard treatment as receipt of surgery followed by adjuvant chemotherapy

^d^Timely initiation of treatment is defined as receipt of a surgery within 30 days of diagnosis

^e^All other includes patients with mental illness/substance abuse with or without physical comorbidities

^f^We identified patients as stably enrolled if they were continuously enrolled in Medicaid ≥ 4 months prior to diagnosis and patients as emergently enrolled if they were continuously enrolled in Medicaid between 3 months before and after the diagnosis

**Fig. 2:**
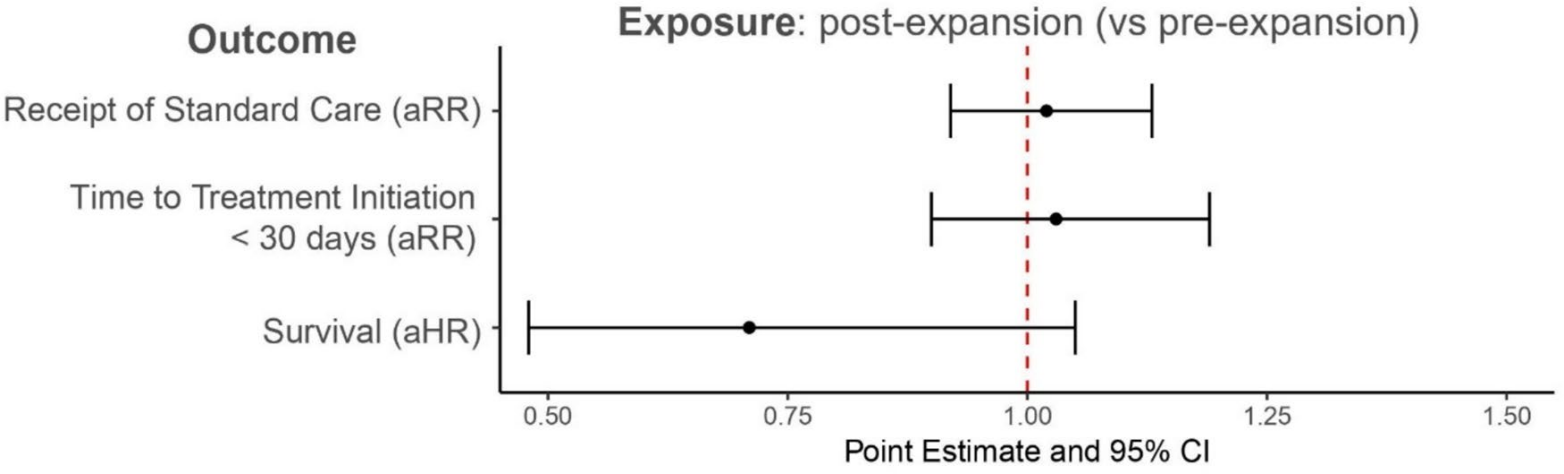
Adjusted association between Medicaid expansion (pre- and post-expansion) and receipt of standard treatment, timely initiation of treatment, and survival among Ohio Medicaid enrollees diagnosed with local or regional colon cancer. Multivariable models for receipt of standard care and timely initiation of treatment adjusted for all individual and area-level characteristics from Table 1. Multivariable model for survival adjusted for all individual- and area-level characteristics in Table 1, except for receipt of treatment. *aRR* adjusted risk ratio, *aHR* adjusted hazard ratio, *CI* confidence interval

Figure 2 presents the adjusted associations between Medicaid expansion (pre- vs. post-expansion) and receipt of standard treatment, timely treatment initiation, and overall survival. We observed adjusted RRs close to 1 with wide confidence intervals, indicating no significant changes in all outcomes in the post-expansion period compared to pre-expansion.

Table 2 presents adjusted associations between individual- and area-level characteristics and each outcome measure, comparing pre-expansion to the two groups in the post-expansion: non-ACA and ACA groups. We observed a trend toward better outcomes in the post-expansion than pre-expansion among those in the ACA group, with slightly higher likelihood of receiving standard treatment (RR 1.09, [0.96, 1.24]) and timely treatment initiation (RR 1.10, [0.93, 1.31]), though these were not statistically significant. Notably, the ACA group in post-expansion showed significantly improved overall survival (HR 0.49, [0.28, 0.88]), indicating a 51% lower risk of death compared to the pre-expansion period. The non-ACA group in post-expansion showed no significant differences in any outcomes compared to pre-expansion.

**Table 2:** Adjusted associations from multivariable models between individual and area-level characteristics with receipt of standard treatment, timely initiation of treatment, and overall survival among Ohio Medicaid enrollees diagnosed with local or regional colon cancer

	Receipt of standard treatment^a^	Timely initiation of treatment^b^	Overall survival^c^
	Risk ratio [95% CI]	Risk ratio [95% CI]	Hazard ratio [95% CI]
Medicaid expansion			
Pre-expansion	REF	REF	REF
ACA group (post-expansion)^d^	1.09	1.10	0.49
	[0.96, 1.24]	[0.93, 1.31]	[0.28, 0.88]
Non-ACA group (post-expansion)^e^	0.97	0.99	0.85
	[0.87, 1.09]	[0.85, 1.16]	[0.56, 1.30]
Timing of enrollment with respect to diagnosis^f^			
Stably enrolled	REF	REF	REF
Emergently enrolled	1.14	1.12	0.94
	[1.02, 1.27]	[0.97, 1.29]	[0.63, 1.39]
Age at diagnosis (years)			
<50	REF	REF	REF
50–54	0.90	0.72	0.96
	[0.79, 1.04]	[0.60, 0.87]	[0.57, 1.63]
55–59	0.82	0.71	0.97
	[0.72, 0.93]	[0.60, 0.84]	[0.60, 1.58]
60–64	0.84	0.75	1.20
	[0.74, 0.96]	[0.63, 0.89]	[0.75, 1.94]
Sex			
Female	REF	REF	REF
Male	0.96	0.98	1.68
	[0.87, 1.05]	[0.86, 1.11]	[1.17, 2.43]
Race/ethnicity			
Non-Hispanic White or other	REF	REF	REF
Non-Hispanic Black	1.01	0.90	0.85
	[0.89, 1.15]	[0.75, 1.07]	[0.53, 1.36]
Marital status			
Not married or unknown	REF	REF	REF
Married	1.10	1.15	0.72
	[0.99, 1.22]	[1.00, 1.32]	[0.47, 1.11]
Stage at diagnosis			
Local	REF	REF	REF
Regional	0.63	1.24	2.43
	[0.58, 0.69]	[1.09, 1.42]	[1.62, 3.63]
Multiple chronic conditions			
No conditions	REF	REF	REF
Physical conditions only	0.98	0.84	1.51
	[0.87, 1.11]	[0.71, 1.00]	[0.90, 2.52]
Other^g^	0.99	0.94	1.80
	[0.87, 1.12]	[0.80, 1.11]	[1.09, 2.98]
*Area-level characteristics*			
Median household income			
Quartile 1 (lowest)	REF	REF	REF
Quartile 2	0.90	1.02	0.98
	[0.79, 1.03]	[0.85, 1.23]	[0.59, 1.64]
Quartile 3	1.04	1.22	0.62
	[0.88, 1.23]	[0.98, 1.53]	[0.32, 1.21]
Quartile 4 (highest)	0.85	1.17	1.63
	[0.67, 1.07]	[0.60, 1.06]	[0.72, 3.65]
Percent of high school diploma among adults aged > 25 years			
Quartile 1 (lowest)	REF	REF	REF
Quartile 2	1.04	0.87	0.52
	[0.91, 1.18]	[0.73, 1.05]	[0.31, 0.86]
Quartile 3	0.85	0.85	0.78
	[0.72, 1.01]	[0.68, 1.06]	[0.41, 1.49]
Quartile 4 (highest)	1.19	0.80	0.30
	[0.96, 1.46]	[0.60, 1.06]	[0.13, 0.73]
Percent of no health insurance among adults aged 19–64 years			
Quartile 1 (lowest)	REF	REF	REF
Quartile 2	0.95	1.19	0.55
	[0.81, 1.13]	[0.96, 1.49]	[0.31, 0.98]
Quartile 3	1.09	1.08	0.46
	[0.89, 1.24]	[0.86, 1.35]	[0.25, 0.86]
Quartile 4 (highest)	1.00	1.08	0.82
	[0.84, 1.19]	[0.85, 1.37]	[0.45, 1.52]
Health professional shortage area			
None	REF	REF	REF
Part or whole	0.94	0.98	1.09
	[0.81, 1.10]	[0.81, 1.19]	[0.63, 1.88]
Rurality of residence			
Appalachia	REF	REF	REF
Non-Appalachia & Non-metro area	0.89	0.98	2.13
	[0.73, 1.07]	[0.76, 1.26]	[1.10, 4.12]
Non-Appalachia & Metro area	1.09	1.08	0.93
	[0.96, 1.24]	[0.91, 1.28]	[0.57, 1.51]

Pre-expansion period includes 2011–2013; post-expansion period includes 2014–2017

^a^Receipt of standard treatment is defined as receipt of standard treatment within 180 days after cancer diagnosis. For local stage, we defined the standard treatment as receipt of surgery. For regional stage, we defined the standard treatment as receipt of surgery followed by adjuvant chemotherapy

^b^Timely initiation of treatment is defined as receipt of a surgery within 30 days of diagnosis

^c^Multivariable model for survival did not adjust for receipt of treatment

^d^ACA group includes Medicaid eligibility categories that were created and became in effect as a result of the ACA in 2014

^e^Non-ACA group includes Medicaid eligibility criteria that were not amended or created as a result of the ACA in 2014

^f^We identified patients as stably enrolled if they were continuously enrolled in Medicaid ≥ 4 months prior to diagnosis and patients as emergently enrolled if they were continuously enrolled in Medicaid between 3 months before and after the diagnosis

^g^All Other includes patients with mental illness/substance abuse with or without physical comorbidities

However, emergently enrolled patients were significantly more likely to receive standard treatment (RR 1.14, [1.02, 1.27]). Older age groups generally showed lower likelihood of receiving standard treatment and timely initiation compared to those <50 years old. Males had significantly higher risk of death compared to females (HR 1.68, [1.17, 2.43]). Regional stage was associated with lower likelihood of standard treatment (RR 0.63, [0.58, 0.69]), higher likelihood of timely initiation (RR 1.24, [1.09, 1.42]), and significantly higher risk of death (HR 2.43, [1.62, 3.63]) compared to local stage. Higher education levels at census tract-level (Quartile 4 of high school diploma) were associated with significantly better survival (HR 0.30, [0.13, 0.73]) compared to the lowest quartile. Non-Appalachia & Non-metro areas showed higher risk of death (HR 2.13, [1.10, 4.12]) compared to Appalachian areas.

Supplementary Table 1 shows significant differences between stably and emergently enrolled individuals, with the former more likely to be enrolled post-expansion (73.7% vs. 58.4%, *p* < 0.001). Emergently enrolled patients were more likely to receive standard treatment (78.7% vs. 70.9%, *p* = 0.046) (Supplementary Table 1). However, our sensitivity analysis that excluded timing of enrollment showed similar magnitudes and statistical significance in the relationship between Medicaid expansion and all outcomes as our main analysis (Supplementary Table 2). Stratified analyses by stage at diagnosis (Supplementary Tables 3 and 4) showed no statistically significant effects of ACA expansion on receipt of standard treatment and timely treatment initiation. However, among regional stage patients, the ACA group in post-expansion showed significantly better survival (HR 0.35, [0.17, 0.72]) and higher likelihood of receiving standard treatment (RR 1.25, [1.05, 1.49]) than pre-expansion (Supplementary Table 4). In comparison to the ACA group, the non-ACA group in the post-expansion period did not differ significantly in any outcomes (Supplementary Table 5).

## Discussions

This study examined the impact of Medicaid expansion on cancer treatment and survival, with a particular focus on two key factors: the timing of Medicaid enrollment relative to cancer diagnosis and the post-expansion Medicaid eligibility criteria, using linked Ohio cancer registry and Medicaid administrative data. We observed that the receipt of standard treatment was similar between pre- and post-expansion periods, with the ACA Group showing a higher, though not statistically significant, rate compared to the Non-ACA Group post-expansion. Timely initiation of treatment showed no significant differences across groups. However, we observed that patients in the post-expansion ACA group had a significantly lower risk of death (51% reduction) than patients in pre-expansion, likely due to their less complex medical needs. Our findings provide important insights for healthcare delivery systems and policymakers regarding how insurance expansion affects cancer care delivery and outcomes. While we observed no significant changes in treatment patterns, the improved survival among ACA-eligible patients suggests that broader insurance coverage may influence outcomes through multiple pathways beyond direct treatment effects, including improved access to supportive care and care coordination.

Several investigations into Medicaid expansion’s influence on cancer care have indicated potential improvements in outcomes. Hoehn et al. found improved surgical care for colon cancer patients in expansion states using NCDB data [15]. Eguia et al. observed an increase in colorectal cancer surgeries in expansion states using HCUP-State Inpatient Data [16]. Sharon et al. reported increased local excisions, decreased emergent surgeries, and reduced 90-day mortality for colorectal cancer patients in expansion states using NCDB data [17]. Gan et al. found improved overall survival among colorectal cancer patients in Kentucky after ACA implementation [8], while Reitz et al. observed improved survival rates for non-metastatic colon cancer patients in expansion states using NCDB data [45]. Despite this growing body of research, few studies utilize the same data sources and linkage methodologies as ours, making direct comparisons challenging. Our results, while generally supportive of the notion that Medicaid expansion improves cancer treatment and survival, show more nuanced outcomes that may differ from previous studies for several reasons. Firstly, our study focuses exclusively on Medicaid-eligible individuals within a single state, unlike multi-state analyses that include both eligible and non-eligible populations. This targeted approach allows us to directly examine expansion’s impact on its intended beneficiaries, revealing that while expansion alone may not improve outcomes, it could be more effective when combined with interventions addressing specific barriers like provider availability and care coordination. This methodological difference helps explain our more modest findings compared to broader studies—while other research may show stronger positive effects by including populations with better baseline access to care and resources, our results reflect the real challenges faced specifically by Medicaid enrollees. Additionally, examining a single-state Medicaid population may show less pronounced due to state-specific factors such as pre-existing healthcare infrastructure, population demographics, and implementation strategies. This distinction highlights that improving outcomes for Medicaid beneficiaries likely requires more comprehensive interventions beyond expansion alone.

We observed that patients who enrolled in Medicaid emergently were more likely to receive standard treatment than patients who had been stably enrolled in Medicaid. Our findings shed light on the complexity of how expanded health insurance coverage via Medicaid may influence cancer management and survival. In our previous work, we noted that patients stably enrolled in Medicaid were more likely to receive an early-stage diagnosis compared to those emergently enrolled [7]. Given this finding, it would be plausible to hypothesize that stable Medicaid enrollment would similarly improve cancer treatment and survival, underlining the importance of enrollment timing on both diagnosis and management [46]. However, in this study, we observed that emergently enrolled patients were more likely to receive standard treatment and initiate treatment within 30 days of diagnosis (though the latter was not statistically significant). This discrepancy suggests that patients noticing abnormal changes or symptoms were more compelled to seek care and subsequently enroll in Medicaid. Indeed, the post-expansion period saw a rise in emergently enrolled patients, particularly from the ACA group, suggesting that Medicaid expansion mitigated financial barriers to accessing treatment for symptomatic individuals without prior health insurance. Our sensitivity analyses, stratifying by cancer stage, underscore the need to further investigate the intricate influence of expanded health insurance on cancer management and survival. These nuanced insights, potentially overlooked in prior studies comparing states with and without Medicaid expansion, highlight the critical role of Medicaid in improving cancer care outcomes.

In addition, patients from non-Appalachian and metro areas had a higher likelihood of receipt of standard treatment and timely treatment initiation than those from Appalachian areas. This aligns with recent findings of higher cancer mortality rates in U.S. counties experiencing persistent poverty, underscoring the need to address underlying socioeconomic factors [47]. The role of persistent poverty should be considered as a possible explanation for this finding given recent evidence suggesting that counties in persistent poverty.

Nonetheless, there are several limitations to this study. First, its single-state focus may limit generalizability, though it provides valuable insights into Medicaid expansion’s impact on cancer treatment and survival. However, we consistently compared patients enrolled in Medicaid between the pre- and post-expansion periods, providing valuable programmatic insights into cancer treatment and survival. Second, our results may be limited by residual and unmeasured confounding. The use of county-level measures rather than more granular geographic data could mask important local variations in healthcare access and resources, and the lack of individual-level socioeconomic factors beyond census tract data may have obscured important social determinants of health that influence treatment patterns and outcomes. Third, we had limited ability to categorize standard treatment accurately or factor in any changes in treatments for colon cancer during the study period, given the high missingness of procedure codes in claims data. Hence, we could only verify patients’ treatment records in the cancer registry and Medicaid claims data to a limited extent. Fourth, the absence of data on patient decision-making and provider recommendations restricts understanding of treatment initiation timing. Since timely treatment is a significant factor associated with increased survival among cancer patients [48], future studies should consider incorporating qualitative information on patients’ decision-making processes regarding cancer treatment and examining provider-physician interpersonal relationships. Fifth, the robustness of our analysis may have been affected by the absence of data on individuals’ health insurance and healthcare utilization history, including historical information on previous health insurance status and healthcare service utilization among patients who newly enrolled in Medicaid post-ACA. We operated under the assumption that some of these patients had prior healthcare interactions, which could lead to conservative estimates of the impact of Medicaid expansion. Lastly, our ability to measure the overall survival of the study population was limited. Incorporating cancer-specific and all-cause mortality data could provide deeper insights into Medicaid expansion’s effects on survival, considering competing health conditions. Nevertheless, we adjusted for the presence of multimorbid conditions to estimate the isolated impact of Medicaid expansion on the survival of our study population.

Despite these constraints, our findings underscore the significance of expanded access to care for improving cancer management and survival. We have also highlighted the nuanced interplay between enrollment mechanisms and cancer care stages, offering insights into patient behaviors and the impact of insurance coverage. These observations contribute valuable insights into how Medicaid expansion affects cancer treatment and survival among Medicaid enrollees diagnosed with non-metastatic colon cancer, utilizing unique and comprehensive data sources.

## Supplementary Information

Below is the link to the electronic supplementary material.

Supplementary file1 (DOCX 71 KB)

## Data Availability

The datasets generated during and/or analyzed during the current study are not publicly available due to Data Use Agreement restrictions with the Ohio Department of Health and the Ohio Department of Medicaid protecting patient privacy and confidential health information.
