# Supplementary material for: Association of Medicaid expansion with colon cancer care: treatment patterns and survival in non-metastatic cases from state registry-claims data
Source: Cancer Causes Control. 2025 Jun 23;36(10):1263–74. doi: 10.1007/s10552-025-01983-8 (PMC12578692; doi:10.1007/s10552-025-01983-8)
Supplement: Supplementary file 1 — Supplementary file1 (DOCX 71 KB) [file 10552_2025_1983_MOESM1_ESM.docx]

**Supplemental Tables:** **Association of Medicaid Expansion with Colon Cancer Care: Treatment Patterns and Survival in Non-Metastatic Cases from State Registry-Claims Data**

**Table of Contents**

S Table 1. Individual- and Area-level Characteristics of Study Population by Timing of Enrollment with respect to Cancer Diagnosis 2

S Table 2. Adjusted Association between Medicaid Expansion and Receipt of Standard Treatment, Timely Initiation of Treatment, and Survival among Ohio Medicaid Enrollees diagnosed with Local or Regional Colon Cancer, excluding Timing of Enrollment 4

S Table 3. Adjusted Associations between Individual and Area-level Characteristics with Receipt of Standard Treatment, Timely Initiation of Treatment, and Survival among Ohio Medicaid Enrollees diagnosed with Local Colon Cancer 5

S Table 4. Adjusted Associations between Individual and Area-level Characteristics with Receipt of Standard Treatment, Timely Initiation of Treatment, and Survival among Ohio Medicaid Enrollees diagnosed with Regional Colon Cancer 8

S Table 5. Adjusted Associations between Individual and Area-level Characteristics with Receipt of Standard Treatment, Timely Initiation of Treatment, and Survival among Ohio Medicaid Enrollees diagnosed with Local or Regional Colon Cancer in the Post-Medicaid Expansion Period 11

# S Table 1. Individual- and Area-level Characteristics of Study Population by Length of Medicaid Enrollment relative to Cancer Diagnosis during the Study Period

|  | **Stably  Enrolled^1^** | **Emergently Enrolled^1^** | **p-value** |
| --- | --- | --- | --- |
| N | 491 | 197 |  |
| Post-Expansion | 362 | 115 | <0.001 |
|  | (73.7) | (58.4) |  |
| Receipt of Standard Treatment^2^ | 348 | 155 | 0.046 |
|  | (70.9) | (78.7) |  |
| *Individual-level Characteristics* |  |  |  |
| Age at Diagnosis |  |  | 0.220 |
| <50 | 110 | 47 |  |
|  | (22.4) | (23.9) |  |
| 51-54 | 91 | 49 |  |
|  | (18.5) | (24.9) |  |
| 55-59 | 145 | 50 |  |
|  | (29.5) | (25.4) |  |
| 60-64 | 145 | 51 |  |
|  | (29.5) | (25.9) |  |
| Male | 239 | 118 | 0.010 |
|  | (48.7) | (59.9) |  |
| Non-Hispanic Black | 127 | 33 | 0.014 |
|  | (25.9) | (16.8) |  |
| Married | 122 | 61 | 0.122 |
|  | (24.8) | (31.0) |  |
| Regional Stage at Diagnosis |  |  | 0.045 |
| Local | 241 | 63 |  |
|  | (41.5) | (32.0) |  |
| Regional | 292 | 134 |  |
|  | (59.5) | (68.0) |  |
| Multiple Chronic Conditions |  |  | <0.001 |
| None | 88 | 64 |  |
|  | (17.9) | (32.5) |  |
| Physical Conditions Only | 192 | 81 |  |
|  | (39.1) | (41.1) |  |
| Other^3^ | 211 | 52 |  |
|  | (43.0) | (26.4) |  |
| *Area-level Characteristics* | | | |
| Household Income below Census-tract Median Level | 334 | 127 | 0.419 |
|  | (68.0) | (64.5) |  |
| High School Diploma among Adults aged >25 years below Census-tract Median Level | 337 | 128 | 0.402 |
|  | (68.6) | (65.0) |  |
| No Health Insurance among Adults aged 19-64 years below Census-tract Median Level | 193 | 86 | 0.335 |
|  | (39.3) | (43.7) |  |
| Part or Whole County in Health Professional Shortage Area | 436 | 158 | 0.004 |
|  | (88.8) | (80.2) |  |
| Residence in Metro Area (county level) | 396 | 149 | 0.173 |
|  | (80.7) | (75.6) |  |
| Frequency (column %) is presented.  Pre-expansion period includes 2011-2013; post-expansion period includes 2014-2017.  ^1^We identified patients as stably enrolled if they were continuously enrolled in Medicaid ≥4 months prior to diagnosis and patients as emergently enrolled if they were continuously enrolled in Medicaid between three months before and after the diagnosis.  ^2^Receipt of Standard Treatment is defined as receipt of standard treatment within 180 days after cancer diagnosis. For local stage, we defined the standard treatment as receipt of surgery. For regional stage, we defined the standard treatment as receipt of surgery followed by adjuvant chemotherapy.  ^3^All Other includes patients with mental illness/substance abuse regardless of physical comorbidities. | | | |

# S Table 2. Adjusted Association between Medicaid Expansion and Receipt of Standard Treatment, Timely Initiation of Treatment, and Survival among Ohio Medicaid Enrollees diagnosed with Local or Regional Colon Cancer, excluding Timing of Enrollment

|  | **Receipt of Standard Treatment^1^** | **Timely Initiation of Treatment^2^** | **Overall Survival^3^** |
| --- | --- | --- | --- |
|  | **Risk Ratio**  **[95% CI]** | **Risk Ratio**  **[95% CI]** | **Hazard Ratio**  **[95% CI]** |
| Pre-Expansion | REF | REF | REF |
| ACA Group  (Post Expansion)^4^ | 1.09 | 1.11 | 0.49 |
|  | [0.96, 1.24] | [0.93, 1.31] | [0.28, 0.88] |
| Non-ACA Group  (Post Expansion)^5^ | 0.94 | 0.97 | 0.86 |
|  | [0.85, 1.05] | [0.83, 1.12] | [0.57, 1.30] |
| Pre-expansion period includes 2011-2013; post-expansion period includes 2014-2017.  ^1^Receipt of Standard Treatment is defined as receipt of standard treatment within 180 days after cancer diagnosis. For local stage, we defined the standard treatment as receipt of surgery. For regional stage, we defined the standard treatment as receipt of surgery followed by adjuvant chemotherapy.  ^2^Time to treatment initiation is defined as receipt of a surgery within 30 days of diagnosis.  ^3^Multivariable model for survival did not adjust for receipt of treatment.  ^4^ACA group includes Medicaid eligibility categories that were created and became in effect as a result of the ACA in 2014.  ^5^Non-ACA group includes Medicaid eligibility criteria that were not amended or created as a result of the ACA in 2014. | | | |

# S Table 3. Adjusted Associations between Individual and Area-level Characteristics with Receipt of Standard Treatment, Timely Initiation of Treatment, and Survival among Ohio Medicaid Enrollees diagnosed with Local Colon Cancer

|  | **Receipt of Standard Treatment^1^** | **Time Initiation of Treatment^2^** | **Overall Survival^3^** |
| --- | --- | --- | --- |
|  | **Risk Ratio**  **[95% CI]** | **Risk Ratio**  **[95% CI]** | **Hazard Ratio**  **[95% CI]** |
| Medicaid Expansion |  |  |  |
| Pre-Expansion | REF | REF | REF |
| ACA Group (Post Expansion)^4^ | 1.04 | 0.93 | 1.47 |
|  | [0.95, 1.14] | [0.66, 1.31] | [0.50, 3.02] |
| Non-ACA Group (Post Expansion)^5^ | 0.96 | 0.83 | 0.68 |
|  | [0.89, 1.04] | [0.61, 1.14] | [0.26, 1.82] |
| Timing of Enrollment^6^ |  |  |  |
| Stably Enrolled | REF | REF | REF |
| Emergently Enrolled | 0.97 | 1.07 | 0.69 |
|  | [0.89, 1.05] | [0.78, 1.47] | [0.27, 1.76] |
| Age at diagnosis (years) |  |  |  |
| <50 | REF | REF | REF |
| 51-54 | 0.94 | 0.48 | 2.28 |
|  | [0.85, 1.05] | [0.31, 0.73] | [0.53, 9.86] |
| 55-59 | 0.91 | 0.67 | 3.33 |
|  | [0.82, 1.01] | [0.47, 0.96] | [0.85, 13.05] |
| 60-64 | 0.97 | 0.66 | 3.55 |
|  | [0.88, 1.06] | [0.47, 0.93] | [0.93, 13.51] |
| Sex |  |  |  |
| Female | REF | REF | REF |
| Male | 0.93 | 0.93 | 1.73 |
|  | [0.87, 1.00] | [0.72, 1.22] | [0.79, 3.77] |
| Race/Ethnicity |  |  |  |
| Non-Hispanic White or Other | REF | REF | REF |
| Non-Hispanic Black | 1.00 | 0.90 | 1.04 |
|  | [0.92, 1.10] | [0.64, 1.27] | [0.40, 2.65] |
| Marital Status |  |  |  |
| Not Married or Unknown | REF | REF | REF |
| Married | 1.05 | 1.13 | 0.29 |
|  | [0.98, 1.14] | [0.84, 1.51] | [0.10, 0.88] |
| Multiple Chronic Conditions |  |  |  |
| No conditions | REF | REF | REF |
| Physical conditions only | 0.98 | 0.79 | 0.31 |
|  | [0.90, 1.07] | [0.56, 1.11] | [0.10, 0.97] |
| Other^7^ | 0.98 | 0.94 | 1.31 |
|  | [0.90, 1.08] | [0.66, 1.32] | [0.48, 3.57] |
| Median Household Income |  |  |  |
| Quartile 1 (lowest) | REF | REF | REF |
| Quartile 2 | 0.97 | 1.14 | 1.24 |
|  | [0.89, 1.07] | [0.80, 1.61] | [0.48, 3.17] |
| Quartile 3 | 1.00 | 1.45 | 2.01 |
|  | [0.88, 1.13] | [0.90, 2.34] | [0.49, 8.25] |
| Quartile 4 (highest) | 0.93 | 1.57 | 1.58 |
|  | [0.77, 1.12] | [0.74, 3.28] | [0.17, 14.39] |
| Percent of High School Diploma among Adults aged >25 years |  |  |  |
| Quartile 1 (lowest) | REF | REF | REF |
| Quartile 2 | 1.11 | 0.87 | 0.21 |
|  | [1.02, 1.21] | [0.62, 1.21] | [0.07, 0.66] |
| Quartile 3 | 1.02 | 0.61 | 0.31 |
|  | [0.90, 1.15] | [0.38, 0.98] | [0.07, 1.29] |
| Quartile 4 (highest) | 1.14 | 0.52 | 0.29 |
|  | [0.96, 1.35] | [0.26, 1.00] | [0.04, 2.07] |
| Percent of No Health Insurance among Adults aged 19-64 years |  |  |  |
| Quartile 1 (lowest) | REF | REF | REF |
| Quartile 2 | 1.11 | 1.16 | 0.85 |
|  | [0.98, 1.26] | [0.72, 1.88] | [0.24, 3.02] |
| Quartile 3 | 1.11 | 0.99 | 0.48 |
|  | [0.98, 1.26] | [0.62, 1.62] | [0.12, 1.90] |
| Quartile 4 (highest) | 1.05 | 0.97 | 1.20 |
|  | [0.93, 1.20] | [0.60, 1.58] | [0.32, 4.52] |
| Health Professional Shortage Area |  |  |  |
| None | REF | REF | REF |
| Part or Whole | 1.01 | 1.01 | 3.44 |
|  | [0.91, 1.14] | [0.67, 1.58] | [0.62, 19.26] |
| Rurality of Residence |  |  |  |
| Appalachia | REF | REF | REF |
| Non-Appalachia & Non-metro area | 0.97 | 0.80 | 7.16 |
|  | [0.85, 1.10] | [0.46, 1.32] | [1.73, 29.63] |
| Non-Appalachia & Metro area | 1.03 | 1.11 | 0.85 |
|  | [0.94, 1.13] | [0.78, 1.58] | [0.31, 2.34] |
| Pre-expansion period includes 2011-2013; post-expansion period includes 2014-2017.  ^1^Receipt of Standard Treatment is defined as receipt of standard treatment within 180 days after cancer diagnosis. For local stage, we defined the standard treatment as receipt of surgery. For regional stage, we defined the standard treatment as receipt of surgery followed by adjuvant chemotherapy.  ^2^Time to treatment initiation is defined as receipt of a surgery within 30 days of diagnosis.  ^3^Multivariable model for survival did not adjust for receipt of treatment.  ^4^ACA group includes Medicaid eligibility categories that were created and became in effect as a result of the ACA in 2014. ^5^Non-ACA group includes Medicaid eligibility criteria that were not amended or created as a result of the ACA in 2014.  ^6^We identified patients as stably enrolled if they were continuously enrolled in Medicaid ≥4 months prior to diagnosis and patients as emergently enrolled if they were continuously enrolled in Medicaid between three months before and after the diagnosis.  ^7^All Other includes patients with mental illness/substance abuse with or without physical comorbidities. | | | |

# S Table 4. Adjusted Associations between Individual and Area-level Characteristics with Receipt of Standard Treatment, Timely Initiation of Treatment, and Survival among Ohio Medicaid Enrollees diagnosed with Regional Colon Cancer

|  | **Receipt of Standard Treatment^1^** | **Time to Treatment Initiation^2^** | **Overall Survival^3^** |
| --- | --- | --- | --- |
|  | **Risk Ratio**  **[95% CI]** | **Risk Ratio**  **[95% CI]** | **Hazard Ratio**  **[95% CI]** |
| Medicaid Expansion |  |  |  |
| Pre-Expansion | REF | REF | REF |
| ACA Group (Post Expansion)^4^ | 1.15 | 1.20 | 0.35 |
|  | [0.93, 1.43] | [0.99, 1.46] | [0.17, 0.72] |
| Non-ACA Group (Post Expansion)^5^ | 0.99 | 1.07 | 0.91 |
|  | [0.81, 1.20] | [0.90, 1.28] | [0.57, 1.47] |
| Timing of Enrollment with respect to diagnosis^6^ |  |  |  |
| Stably Enrolled | REF | REF | REF |
| Emergently Enrolled | 1.25 | 1.11 | 1.11 |
|  | [1.05, 1.49] | [0.94, 1.30] | [0.71, 1.75] |
| Age at diagnosis (years) |  |  |  |
| <50 | REF | REF | REF |
| 51-54 | 0.92 | 0.83 | 0.85 |
|  | [0.73, 1.15] | [0.68, 1.02] | [0.47, 1.53] |
| 55-59 | 0.77 | 0.71 | 0.74 |
|  | [0.62, 0.96] | [0.58, 0.86] | [0.43, 1.29] |
| 60-64 | 0.75 | 0.77 | 1.08 |
|  | [0.60, 0.94] | [0.63, 0.93] | [0.63, 1.85] |
| Sex |  |  |  |
| Female | REF | REF | REF |
| Male | 1.00 | 0.99 | 1.64 |
|  | [0.85, 1.18] | [0.86, 1.15] | [1.07, 2.51] |
| Race/Ethnicity |  |  |  |
| Non-Hispanic White or Other | REF | REF | REF |
| Non-Hispanic Black | 1.03 | 0.90 | 0.76 |
|  | [0.83, 1.28] | [0.73, 1.11] | [0.44, 1.32] |
| Marital Status |  |  |  |
| Not Married or Unknown | REF | REF | REF |
| Married | 1.21 | 1.18 | 0.87 |
|  | [1.01, 1.45] | [1.00, 1.39] | [0.54, 1.40] |
| Multiple Chronic Conditions |  |  |  |
| No conditions | REF | REF | REF |
| Physical conditions only | 0.95 | 0.85 | 2.04 |
|  | [0.76, 1.18] | [0.70, 1.04] | [1.10, 3.78] |
| Other^7^ | 0.95 | 0.92 | 1.85 |
|  | [0.76, 1.19] | [0.76, 1.12] | [1.00, 3.43] |
| Median Household Income |  |  |  |
| Quartile 1 (lowest) | REF | REF | REF |
| Quartile 2 | 0.86 | 0.98 | 0.83 |
|  | [0.67, 1.10] | [0.78, 1.22] | [0.43, 1.57] |
| Quartile 3 | 1.12 | 1.20 | 0.45 |
|  | [0.84, 1.49] | [0.93, 1.54] | [0.20, 1.02] |
| Quartile 4 (highest) | 0.84 | 1.09 | 1.49 |
|  | [0.57, 1.22] | [0.78, 1.51] | [0.61, 3.63] |
| Percent of High School Diploma among Adults aged >25 years |  |  |  |
| Quartile 1 (lowest) | REF | REF | REF |
| Quartile 2 | 0.95 | 0.89 | 0.60 |
|  | [0.75, 1.21] | [0.72, 1.10] | [0.32, 1.10] |
| Quartile 3 | 0.71 | 0.95 | 0.96 |
|  | [0.52, 0.96] | [0.73, 1.23] | [0.44, 2.12] |
| Quartile 4 (highest) | 1.13 | 0.95 | 0.25 |
|  | [0.80, 1.58] | [0.79, 1.29] | [0.09, 0.74] |
| Percent of No Health Insurance among Adults aged 19-64 years |  |  |  |
| Quartile 1 (lowest) | REF | REF | REF |
| Quartile 2 | 0.84 | 1.22 | 0.49 |
|  | [0.64, 1.11] | [0.96, 1.56] | [0.25, 0.96] |
| Quartile 3 | 1.00 | 1.13 | 0.42 |
|  | [0.76, 1.32] | [0.87, 1.46] | [0.20, 0.86] |
| Quartile 4 (highest) | 0.89 | 1.14 | 0.75 |
|  | [0.66, 1.19] | [0.87, 1.50] | [0.37, 1.53] |
| Health Professional Shortage Area |  |  |  |
| None | REF | REF | REF |
| Part or Whole | 0.86 | 0.97 | 0.97 |
|  | [0.67, 1.11] | [0.79, 1.22] | [0.52, 1.82] |
| Rurality of Residence |  |  |  |
| Appalachia | REF | REF | REF |
| Non-Appalachia & Non-metro area | 0.80 | 1.10 | 1.62 |
|  | [0.55, 1.14] | [0.82, 1.47] | [0.71, 3.71] |
| Non-Appalachia & Metro area | 1.18 | 1.08 | 0.90 |
|  | [0.95, 1.47] | [0.89, 1.31] | [0.51, 1.60] |
| Pre-expansion period includes 2011-2013; post-expansion period includes 2014-2017.  ^1^Receipt of Standard Treatment is defined as receipt of standard treatment within 180 days after cancer diagnosis. For local stage, we defined the standard treatment as receipt of surgery. For regional stage, we defined the standard treatment as receipt of surgery followed by adjuvant chemotherapy.  ^2^Timely Initiation of Treatment is defined as receipt of a surgery within 30 days of diagnosis.  ^3^Multivariable model for survival did not adjust for receipt of treatment.  ^4^ACA group includes Medicaid eligibility categories that were created and became in effect as a result of the ACA in 2014. ^5^Non-ACA group includes Medicaid eligibility criteria that were not amended or created as a result of the ACA in 2014.  ^6^We identified patients as stably enrolled if they were continuously enrolled in Medicaid ≥4 months prior to diagnosis and patients as emergently enrolled if they were continuously enrolled in Medicaid between three months before and after the diagnosis.  ^7^All Other includes patients with mental illness/substance abuse with or without physical comorbidities. | | | |

# S Table 5. Adjusted Associations between Individual and Area-level Characteristics with Receipt of Standard Treatment, Timely Initiation of Treatment, and Survival among Ohio Medicaid Enrollees diagnosed with Local or Regional Colon Cancer in the Post-Medicaid Expansion Period

|  | **Receipt of Standard Treatment^1^** | **Time to Treatment Initiation^2^** | **Overall Survival^3^** |
| --- | --- | --- | --- |
|  | **Risk Ratio**  **[95% CI]** | **Risk Ratio**  **[95% CI]** | **Hazard Ratio**  **[95% CI]** |
| Medicaid Expansion |  |  |  |
| ACA Group^4^ | REF | REF | REF |
| Non-ACA Group^5^ | 0.90 | 0.94 | 1.85 |
|  | [0.79, 1.02] | [0.79, 1.11] | [0.96, 3.57] |
| Timing of Enrollment with respect to diagnosis^6^ |  |  |  |
| Stably Enrolled | REF | REF | REF |
| Emergently Enrolled | 1.17 | 1.19 | 1.01 |
|  | [1.02, 1.34] | [0.99, 1.43] | [0.51, 2.00] |
| Age at diagnosis (years) |  |  |  |
| <50 | REF | REF | REF |
| 51-54 | 0.94 | 0.65 | 0.99 |
|  | [0.79, 1.12] | [0.51, 0.82] | [0.46, 2.13] |
| 55-59 | 0.83 | 0.73 | 0.90 |
|  | [0.70, 0.98] | [0.59, 0.90] | [0.44, 1.81] |
| 60-64 | 0.86 | 0.70 | 0.62 |
|  | [0.73, 1.01] | [0.57, 0.87] | [0.29, 1.32] |
| Sex |  |  |  |
| Female | REF | REF | REF |
| Male | 0.99 | 1.04 | 1.36 |
|  | [0.88, 1.11] | [0.89, 1.22] | [0.79, 2.34] |
| Race/Ethnicity |  |  |  |
| Non-Hispanic White or Other | REF | REF | REF |
| Non-Hispanic Black | 1.02 | 0.94 | 0.55 |
|  | [0.87, 1.20] | [0.75, 1.18] | [0.25, 1.19] |
| Marital Status |  |  |  |
| Not Married or Unknown | REF | REF | REF |
| Married | 1.06 | 1.15 | 0.88 |
|  | [0.93, 1.20] | [0.97, 1.36] | [0.47, 1.64] |
| Stage at Diagnosis |  |  |  |
| Local | REF | REF | REF |
| Regional | 0.63 | 1.35 | 2.29 |
|  | [0.57, 0.71] | [1.14, 1.59] | [1.23, 4.24] |
| Multiple Chronic Conditions |  |  |  |
| No conditions | REF | REF | REF |
| Physical conditions only | 0.94 | 0.93 | 2.60 |
|  | [0.81, 1.09] | [0.76, 1.14] | [1.03, 6.59] |
| Other^7^ | 0.92 | 0.98 | 2.68 |
|  | [0.79, 1.08] | [0.79, 1.21] | [1.06, 6.77] |
| Median Household Income |  |  |  |
| Quartile 1 (lowest) | REF | REF | REF |
| Quartile 2 | 0.91 | 0.98 | 0.86 |
|  | [0.77, 1.07] | [0.78, 1.24] | [0.41, 1.84] |
| Quartile 3 | 1.04 | 1.29 | 0.28 |
|  | [0.85, 1.28] | [0.98, 1.69] | [0.09, 0.87] |
| Quartile 4 (highest) | 0.87 | 1.12 | 1.12 |
|  | [0.65, 1.15] | [0.77, 1.61] | [0.33, 3.78] |
| Percent of High School Diploma among Adults aged >25 years |  |  |  |
| Quartile 1 (lowest) | REF | REF | REF |
| Quartile 2 | 1.00 | 0.88 | 0.35 |
|  | [0.86, 1.17] | [0.70, 1.09] | [0.15, 0.83] |
| Quartile 3 | 0.80 | 0.84 | 0.99 |
|  | [0.65, 0.98] | [0.64, 1.10] | [0.39, 2.47] |
| Quartile 4 (highest) | 1.08 | 0.74 | 0.26 |
|  | [0.83, 1.39] | [0.53, 1.04] | [0.07, 0.95] |
| Percent of No Health Insurance among Adults aged 19-64 years |  |  |  |
| Quartile 1 (lowest) | REF | REF | REF |
| Quartile 2 | 0.87 | 1.16 | 0.65 |
|  | [0.71, 1.08] | [0.89, 1.51] | [0.27, 1.57] |
| Quartile 3 | 1.05 | 1.06 | 0.35 |
|  | [0.86, 1.29] | [0.81, 1.40] | [0.13, 0.92] |
| Quartile 4 (highest) | 0.96 | 0.99 | 0.41 |
|  | [0.78, 1.19] | [0.74, 1.32] | [0.15, 1.10] |
| Health Professional Shortage Area |  |  |  |
| None | REF | REF | REF |
| Part or Whole | 0.97 | 0.90 | 1.29 |
|  | [0.81, 1.17] | [0.71, 1.15] | [0.54, 3.10] |
| Rurality of Residence |  |  |  |
| Appalachia | REF | REF | REF |
| Non-Appalachia & Non-metro area | 0.83 | 1.04 | 1.32 |
|  | [0.65, 1.03] | [0.77, 1.4] | [0.47, 3.74] |
| Non-Appalachia & Metro area | 0.98 | 1.08 | 0.70 |
|  | [0.85, 1.14] | [0.89, 1.33] | [0.37, 1.33] |
| Pre-expansion period includes 2011-2013; post-expansion period includes 2014-2017.  ^1^Receipt of Standard Treatment is defined as receipt of standard treatment within 180 days after cancer diagnosis. For local stage, we defined the standard treatment as receipt of surgery. For regional stage, we defined the standard treatment as receipt of surgery followed by adjuvant chemotherapy.  ^2^Timely Initiation of Treatment is defined as receipt of a surgery within 30 days of diagnosis.  ^3^Multivariable model for survival did not adjust for receipt of treatment.  ^4^ACA group includes Medicaid eligibility categories that were created and became in effect as a result of the ACA in 2014. ^5^Non-ACA group includes Medicaid eligibility criteria that were not amended or created as a result of the ACA in 2014.  ^6^We identified patients as stably enrolled if they were continuously enrolled in Medicaid ≥4 months prior to diagnosis and patients as emergently enrolled if they were continuously enrolled in Medicaid between three months before and after the diagnosis.  ^7^All Other includes patients with mental illness/substance abuse with or without physical comorbidities. | | | |
